# O15 Recognition of subclinical macrophage activation syndrome in an adolescent systemic juvenile idiopathic arthritis patient receiving tocilizumab: a case report

**DOI:** 10.1093/rap/rkaa054.003

**Published:** 2020-11-03

**Authors:** Sue Ng, Jonathan Talbot, Jacqui Clinch, Vicky Ohlsson, Valerie Rogers

**Affiliations:** r1 Paediatric Rheumatology; Bristol Royal Hospital for Children, Bristol, United Kingdom; r2 Paediatrics; Derriford Hospital, Plymouth, United Kingdom

## Abstract

**Case report - Introduction:**

Macrophage Activation Syndrome (MAS) affects approximately 10% of patients with Systemic Juvenile Idiopathic Arthritis (sJIA) which left untreated can have fatal consequences. MAS in sJIA often occurs in the context of uncontrolled disease or following a concurrent infectious trigger. Overactive T lymphocytes and macrophages produce a cytokine storm of pro-inflammatory cytokines including TNF-α, IL-1β, IL-6, IL-18, IFN-γ and IFN-γ induced chemokines. Tocilizumab (TCZ), an anti-IL6 receptor monoclonal antibody, can be used in the treatment of sJIA. Research has shown that patients receiving Biological therapies may have fewer symptoms and less pronounced biochemical changes than Biological-naïve patients at presentation with MAS.

**Case report - Case description:**

We highlight the case of a 12-year-old female diagnosed with sJIA complicated by MAS. Her initial presentation fulfilled the 2016 EULAR/ACR criteria for MAS in sJIA with daily fever, rash, hyperferritinemia (19058 ng/ml), hypertriglyceridemia (362 mg/dl) and raised aspartate aminotransferase (230 U/l) with a marrow demonstrating haemophagocytosis. She was treated with pulsed Methylprednisolone (MP), oral Prednisolone (PNL), and fortnightly IV Tocilizumab 8 mg/kg. Once controlled, she switched to weekly subcutaneous Tocilizumab 162 mg and weaned Prednisolone.

Several months after diagnosis, following a tooth abscess and shingles, the patient represented with intermittent fever, pruritic rash, myalgia, generalised arthralgia, and a rapid onset of enlarging organomegaly. MRI identified cervical, axillary, and inguinal lymphadenopathy and marked hepatosplenomegaly (Spleen 17 cm). Lymphoproliferative disorders were excluded through marrow aspiration and lymph node biopsy. During this presentation, the biochemistry did not meet the 2016 EULAR/ACR criteria. However, she was treated for suspected MAS due to the Tocilizumab therapy. After switching from Tocilizumab to subcutaneous Anakinra (ANA) 2 mg/kg/d with three days IV Methylprednisolone 30 mg/kg, her symptoms and biochemical parameters improved (Table 1).

**Case report - Discussion:**

IL-6 is a key player of immune activation and is involved in the generation of the acute phase response. The use of Tocilizumab, an IL-6 inhibitor, masks the febrile picture of MAS and it is therefore unsurprising to see an altered representation of MAS biochemically. Alterations seen in this case study included a delayed rise in acute phase reactants (ESR, CRP), with absence of hyperferritinemia and hypertriglyceridemia. However, the ongoing hemophagocytic process with increased cell turnover within the reticuloendothelial system still caused marked organomegaly with significant biochemical changes seen via a steadily rising LDH and transaminases. Biological treatment for sJIA therefore alters the traditional clinical presentation and inflammatory biochemical profile resulting in patients who may not necessarily fulfil the 2016 EULAR/ACR criteria for MAS in sJIA.

Functional assays were performed looking at the perforin-cytolytic pathway during both MAS presentations (Table 1). The first test during the initial diagnosis of sJIA reported a defective granular release assay and perforin deficiency, but a relative normal sCD25. However, this followed treatment with steroids. The latter test showed normal expression of both NK cell and perforin. The normal repeat functional assays following a year of Tocilizumab makes a genetic aetiology unlikely. This suggests an acquired form of a functional deficiency of both NK cells and perforin activity, with dysfunction of both, can be seen in the background of active sJIA. This function is often restored once systemic inflammation is controlled. Infectious aetiology for Leishmania was considered but as the histology of the marrow lacks histiocytes infiltration, no further DNA PCR testing was offered.

**Case report - Key learning points:**

The occurrence of MAS in sJIA is often presumed in the context of a background of uncontrolled inflammation or following concurrent infectious triggers. The use of biologics with cytokine directed therapies have revolutionized the treatment of sJIA and remarkably shortened the time to achieve inactive disease. In this case study we learn that achieving disease control does not necessarily prevent MAS as this complex phenomenon has multiple aetiologies which drive the pathway towards a cytokine storm.

This case study as well as data from phase III clinical trials of Tocilizumab, report that treatment using Tocilizumab does not confer protection against the development of MAS. This observation concludes that subtle deterioration of a sJIA patient receiving biologics should prompt additional investigation to exclude a subclinical MAS presentation. Absence of fever and a normal ferritin level does not negate the possibility of a partially treated MAS. Patients receiving Tocilizumab, an IL-6 inhibitor, are less likely to be febrile, and have lower ferritin, CRP, and triglycerides levels, thus making interpretation of MAS biochemical results difficult.

This indirectly validates the presence of other cytokines orchestrating the pathogenesis of MAS in the context of a systemically controlled sJIA on IL-6 inhibitor. Emerging evidence have reported disproportionately elevated levels of circulating IL-18, IFN-γ and IFN-γ chemokines in sJIA patients who develop MAS.

This case highlights the importance of clinicians retaining a high degree of suspicion of a ‘subclinical MAS’ in children on cytokine blockade therapies. Further prospective validation of the MAS in sJIA 2016 EULAR/ACR criteria is needed as there are clearly limitation of its use in patients on biologics. Potentially a separate criterion is needed to diagnose MAS in sJIA patients on Tocilizumab to avoid underdiagnosis of this potentially fatal condition.

**O15 Table 1:** Investigations and Management over admission. Granule Release and Perforin Studies.

Day of Admission	D1	D3	D5	D8	
Ferritin(ng/ml)	469	574	610	169	
Platelets(10^9^/l)	297	253	232	381	
AST(U/l)	90		124	53	
Triglycerides(mg/dl)		398	310	460	
Fibrinogen(g/l)	230	210	220	120	
CRP (mg/l)	16	5	5	0	
LDH(U/l)	1200	1409	2591	1329	
Treatment	SC TCZ 162 mg/wk		IV MP + ANA	PNL+ANA	
**Investigation**				**Pre-Treatment**	**Post-Treatment**
Granule Release Assay	Control *vs* Patient CD3 (CD8+CD107A+) %			0.6 / 0.1	0.4 / 0.4
	Control *vs* Patient PHA (NK cellsCD107a+) %			11.0 / 0.0	2.0 / 5.6
Perforin Assay	Control *vs* Patient Perforin (CD56+cells) %			74.5 / 30.6	72.8 / 83.3
Comment				Abnormal cytotoxic granule release assay.	Normal cytotoxic granule release assay.
				Abnormal perforin expression.	Normal perforin expression.

